# Time-controllable *Nkcc1* knockdown replicates reversible hearing loss in postnatal mice

**DOI:** 10.1038/s41598-017-13997-7

**Published:** 2017-10-19

**Authors:** Takahisa Watabe, Ming Xu, Miho Watanabe, Junichi Nabekura, Taiga Higuchi, Karin Hori, Mitsuo P. Sato, Fumiaki Nin, Hiroshi Hibino, Kaoru Ogawa, Masatsugu Masuda, Kenji F. Tanaka

**Affiliations:** 1 Department of Otolaryngology, Keio University School of Medicine, 35 Shinanomachi, Shinjuku-ku, Tokyo, 160-8582 Japan; 2 Department of Neuropsychiatry, Keio University School of Medicine, 35 Shinanomachi, Shinjuku-ku, Tokyo, 160-8582 Japan; 3 Department of Neurophysiology, Hamamatsu University School of Medicine, 1-20-1 Handayama, Higashi-ku, Hamamatsu city, Shizuoka, 431-3192 Japan; 4 Division of Homeostatic Development, National Institute for Physiological Sciences, 38 Nishigonaka Myodaiji, Okazaki, Aichi 444-8585 Japan; 5 Department of Molecular Physiology, Niigata University School of Medicine, 757 Ichibancho, Asahimachi-dori, Chuo-ku, Niigata-shi, Niigata, 951-8510 Japan; 6 Center for Transdisciplinary Research, Niigata University, 8050 Ikarashi 2-no-cho, Nishi-ku, Niigata, 950-2181 Japan; 7 Department of Otolaryngology, Kyorin University School of Medicine, 6-20-2 Shinkawa, Mitaka-shi, Tokyo, 181-8611 Japan

## Abstract

Identification of the causal effects of specific proteins on recurrent and partially reversible hearing loss has been difficult because of the lack of an animal model that provides reversible gene knockdown. We have developed the transgenic mouse line *Actin*-tTS::*Nkcc1* ^tetO/tetO^ for manipulatable expression of the cochlear K^+^ circulation protein, NKCC1. *Nkcc1* transcription was blocked by the binding of a tetracycline-dependent transcriptional silencer to the tetracycline operator sequences inserted upstream of the *Nkcc1* translation initiation site. Administration of the tetracycline derivative doxycycline reversibly regulated *Nkcc1* knockdown. Progeny from pregnant/lactating mothers fed doxycycline-free chow from embryonic day 0 showed strong suppression of *Nkcc1* expression (~90% downregulation) and *Nkcc1* null phenotypes at postnatal day 35 (P35). P35 transgenic mice from mothers fed doxycycline-free chow starting at P0 (delivery) showed weaker suppression of *Nkcc1* expression (~70% downregulation) and less hearing loss with mild cochlear structural changes. Treatment of these mice at P35 with doxycycline for 2 weeks reactivated *Nkcc1* transcription to control levels and improved hearing level at high frequency; i.e., these doxycycline-treated mice exhibited partially reversible hearing loss. Thus, development of the *Actin*-tTS::*Nkcc1* ^tetO/tetO^ transgenic mouse line provides a mouse model for the study of variable hearing loss through reversible knockdown of *Nkcc1*.

## Introduction

**Figure 1:**
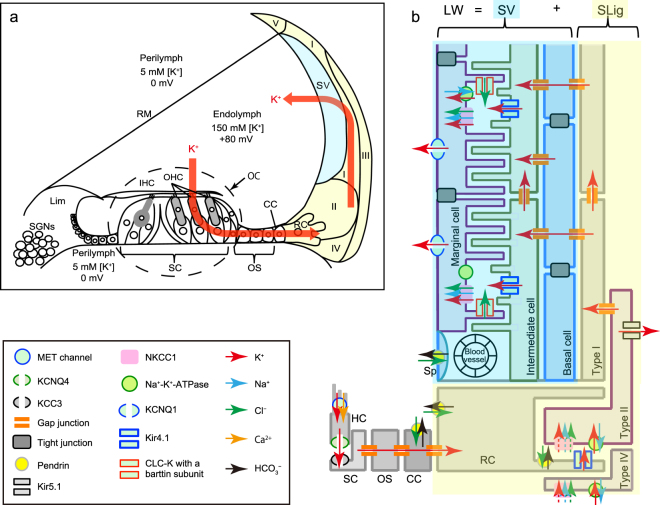
Overview of cochlear structures and the K^+^ recycling pathway through the LW. (**a**) K^+^ flows into the inner and outer hair cells (IHC and OHC) in the organ of Corti (OC) from the high K^+^ endolymph in the scala media during auditory transduction. K^+^ is transmitted through supporting cells (SC), outer sulcus cells (OS) including Claudius cells (CC), root cells (RC), type II and type I fibrocytes in the SLig, and cells in the SV, and then returns to the endolymph. This recycling pathway is critical for maintaining cochlear lymphatic homeostasis and generating EP, and for normal hearing. Lim: spiral limbus; RM, Reissner’s membrane; (**b**) Various K^+^ circulation molecules work cooperatively to maintain cochlear lymphatic homeostasis. NKCC1 in the marginal cells is essential for the K^+^ recycling necessary for normal hearing, but it is functionally silent for K^+^ recycling in the SLig. As NKCC1 does not contribute to normal hearing in the SLig cells^27^, ion flows are shown by dashed arrows. MET: mechanoelectrical transduction; Sp, spindle cell.

Although the mechanisms underlying recurrent and partially reversible hearing loss (variable hearing loss) are unknown, the symptoms that appear in patients with various types of sensorineural hearing loss (SNHL) like idiopathic acute sensorineural hearing loss, fluctuating low-tone SNHL, and Ménière’s disease^1–5^ are very typical. Reports have suggested the lateral wall (LW) of the cochlea as a candidate for the pathogenetic site that causes such variable hearing loss^1,6–10^. As the main route for potassium (K^+^) recycling in the cochlea, the LW is critical to the generation of the endocochlear potential (EP) and thus for normal hearing (Fig. 1a,b)^11–15^.

The LW consists of the stria vascularis (SV) and the spiral ligament (SLig). The SV consists of three types of stratified cells, namely the marginal, the intermediate, and the basal cells (Fig. 1b). In the SLig, five types of fibrocytes are found preferentially in different sites. These cells show specific expression profiles for proteins that function in K^+^ circulation and contribute to unidirectional K^+^ recycling. The well-controlled high K^+^ endolymph generates a large driving force for K^+^ influx into sensory hair cells (HCs), which transduce the mechanical energy of sound to electrical energy for auditory neurons^11–14^. Thus, analyses of K^+^ transport mechanisms are important for the clarification of the molecular pathophysiological mechanisms of hearing loss.

The Na^+^/K^+^/2Cl^−^ co-transporter (NKCC1) is one of a number of proteins that function in K^+^ circulation in the cochlea^11,16–20^. It is encoded by solute carrier family 12 member 2 (*Slc12a2*) in mouse (we refer to *Slc12a2* as its synonym, *Nkcc1*, hereafter to avoid confusion with *Slc26a4 that* encodes pendrin). In general, NKCC1 regulates cellular osmolality, cellular volume, and fluid transport^21^. In the postnatal mammalian cochlea, NKCC1 is expressed by the basolateral membrane of the marginal cells, type II, IV, and V fibrocytes in the SLig, fibrocytes in the spiral limbus (Lim), and satellite cells surrounding spiral ganglion neurons (SGNs)^22–26^. The importance of NKCC1 for normal hearing was demonstrated by loss-of-function manipulations such as the disruption of *Nkcc1* ^16,18–20^ or the systemic administration of NKCC1 inhibitors^17,27^.

**Figure 2:**
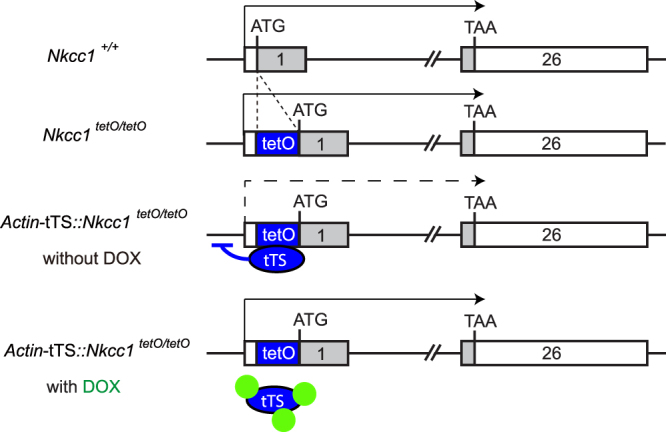
Genetic elements of the doxycycline-regulated *Nkkc1* suppression system. The construct for *Actin*-tTS::*Nkcc1* ^tetO/tetO^. *Nkcc1* consists of 26 exons. The translation initiation site (ATG) is situated at exon 1, and the stop site marks the end of translation at exon 26. The tetracycline operator site (tetO) was inserted just upstream of ATG to form *Nkcc1* ^tetO/tetO^. *Nkcc1* ^tetO/tetO^ homozygotes behave like wild-type mice. The tetracycline trans-silencer (tTS) tethers tetO in the absence of doxycycline (DOX), resulting in the suppression of *Nkcc1* expression. In the presence of DOX (green circles), tTS-mediated gene knockdown does not occur. Gray boxes: coding regions in exons 1 and 26.

Regardless of the number of studies that have focused on the roles of the various molecules involved in cochlear K^+^ circulation, the pathogenesis of variable hearing loss remains unknown. There are few animal models for which expression of an auditory-related gene of interest can be repeatedly manipulated at will—in particular, to model variable hearing loss. Conventional gene knockout in mice is a useful tool, but temporal control of loss and reactivation of gene expression is not applicable. Pharmacological approaches are beneficial in terms of acute intervention, but this approach is not always suitable in cases of chronic and stable loss of function because of side effects induced by high dosage or nonspecific effects. To overcome these drawbacks, we employed the tetracycline-regulated gene suppression system in which administration and subsequent discontinuation of the tetracycline derivative doxycycline (DOX) controls suppression of a target gene (Fig. 2)^28,29^. This system requires two distinct transgenic mouse lines: one that expresses the tetracycline-dependent transcriptional silencer (tTS), and a second, a knock-in line, that carries the tetracycline operator site (tetO) upstream of the gene of interest. In transgenic mice carrying both constructs, tTS tethers the tetO sites (of both alleles) and blocks transcription of the target gene. Formation of the repressor tTS/tetO complex is reversibly inhibited by DOX binding to the tTS; i.e., DOX relieves suppression of transcription.

In the present report, we generated tetO knock-in mice at the *Nkcc1* locus and crossed them with the tTS-expressing line, *Actin*-tTS^29^, to establish the double transgenic line, *Actin*-tTS::*Nkcc1* ^tetO/tetO^, that we used for tTS-mediated *Nkcc1* knockdown. We demonstrate inducible and reversible *Nkcc1* knockdown by DOX administration and discontinuation. By taking advantage of the tTS-tetO technology, we assessed whether postnatal *Nkcc1* knockdown could cause hearing loss and if such hearing loss could be rescued by gene reactivation in *Actin*-tTS::*Nkcc1* ^tetO/tetO^ mice.

## Results

### Establishment of tTS-mediated *Nkcc1* knockdown mouse line

To establish tTS-mediated *Nkcc1* knockdown, we first generated *Nkcc1* tetO knock-in mice with the Flexible Accelerated STOP tetO-knock-in (FAST) system^28^. The cassette containing PGK-Neo, STOP sequence, and tetO site was inserted just upstream of the translation initiation site of *Nkcc1* within exon 1; subsequently, Neo-STOP was excised by Flippase-mediated recombination, to yield *Nkcc1* tetO knock-in mice (*Nkcc1* ^tetO^; Supplementary Fig. S1). We crossed *Nkcc1* ^tetO^ mice with *Actin*-tTS transgenic mice^29^ (ubiquitous tTS-expressing line) and obtained *Actin*-tTS hemizygous, *Nkcc1* ^tetO^ homozygous mice (*Actin*-tTS::*Nkcc1* ^tetO/tetO^; Fig. 2). The genetic background was mixed 129S6/C57BL6J.

Theoretically, in the absence of DOX (i.e., normal chow feed for pregnant and lactating mothers), *Nkcc1* expression would be disrupted throughout the entire lifetime of *Actin* tTS::*Nkcc1* ^tetO/tetO^ mice embryos and pups, which would induce a *Nkcc1* null phenotype equivalent to that of conventional *Nkcc1* knockout mice^18,20^. We estimated the gross phenotype by measuring the body weight of mice and evaluating their posture. *Actin*-tTS::*Nkcc1* ^tetO/tetO^ mice showed apparent growth retardation (n = 6; average body weight 13.9 ± 3.1 g) compared with their *Nkcc1* ^tetO/tetO^ littermates as controls (n = 6; 17.8 ± 0.7 g), although the difference was not significant (*p* = 0.0649, Mann-Whitney test). Interestingly, half of *Actin*-tTS::*Nkcc1* ^tetO/tetO^ mice started tilting, circling, or head bobbing at P17–18, and those postural perturbations lasted throughout adulthood. The remainder of the mice did not show obvious postural perturbations. The juvenile onset of postural perturbations was consistent with the vestibular abnormality seen in conventional *Nkcc1* null mutants.

**Figure 3:**
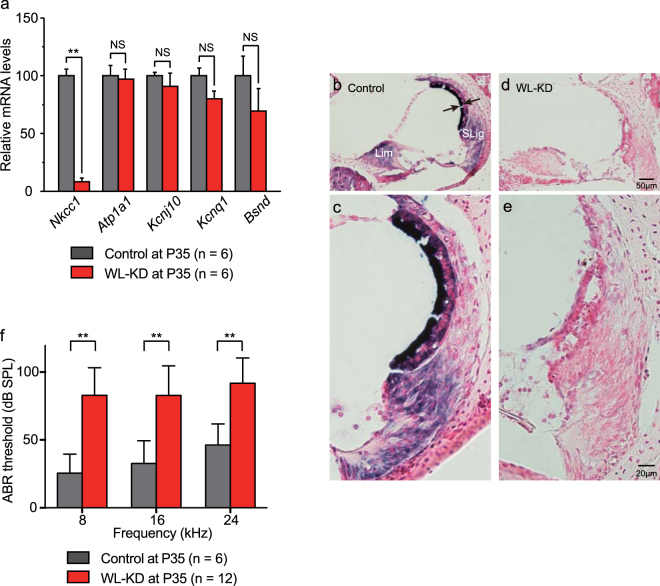
Quantitative, qualitative, and functional analyses of lifetime *Nkcc1* knockdown effects in *Actin*-tTS::*Nkcc1* ^tetO/tetO^ mice (WL-KD mice). (**a**) At P35, cochlear *Nkcc1* transcription is decreased in WL-KD mice vs. controls in comparison with other K^+^ circulation molecules as labeled. mRNA levels are normalized to *Gapdh*. *Nkcc1* mRNA was significantly suppressed in WL-KD mice compared with controls, but the mRNAs of other K^+^ circulation molecules were not suppressed. (**b**–**e**) *In situ* hybridization of *Nkcc1* mRNA in mouse cochleae. (**b**) Normal *Nkcc1* mRNA expression in cochlea of control. Arrows show normal SV thickness. (**c**) Magnification of view in (**b**). (**d**) Strong suppression of *Nkcc1* level in WL-KD mice. (**e**) Magnification of view in (**d**). (**f**) *Nkcc1* knockdown significantly elevates the ABR threshold, which indicates hearing loss. **Significant difference at *p* < 0.01 (Mann-Whitney test). NS: no significant difference (Mann-Whitney test).

To confirm tTS-mediated gene knockdown, we quantified the cochlear *Nkcc1* mRNA level. We isolated total RNA from cochlea (n = 6, for each genotype) of *Actin*-tTS::*Nkcc1* ^tetO/tetO^ and controls at P35. We conducted quantitative reverse transcription-PCR (qRT-PCR) for *Nkcc1* and other cochlear genes encoding K^+^ circulation molecules including *Atp1a1* (encoding α1-Na^+^/K^+^-ATPase), *Kcnj10* (Kir4.1), *Kcnq1* (KCNQ1), and *Bsnd* (Barttin). The *Nkcc1* mRNA level was significantly decreased to a level of 10.4% compared with that of controls (*p* = 0.0022, Mann-Whitney test; Fig. 3a), but no significant differences were found in mRNA levels of the other K^+^ circulation molecules. These results validated specific *Nkcc1* knockdown by the tTS-tetO system in the absence of DOX.

We then analyzed *Nkcc1* knockdown histologically. RNA *in situ* hybridization was performed on cross-sections of the cochlea isolated from mice at ≥P21. In controls, *Nkcc1* was strongly expressed in the SV (arrows in Fig. 3b) and moderately expressed in type II, IV, and V fibrocytes in the SLig, fibrocytes in the Lim, and satellite cells surrounding the SGNs, as reported^22–24^ (Fig. 3b,c). *Nkcc1* RNA labeling was observed throughout the full thickness of the SV from the nearby nucleus to the periphery of the interior processes of the marginal cells. In *Actin*-tTS::*Nkcc1* ^tetO/tetO^ cochlea of mice without DOX treatment, *Nkcc1* mRNA was not detectable (Fig. 3d,e), which is consistent with the qRT-PCR results.

As reported, lifetime *Nkcc1* disruption causes hearing loss^18–20^. To quantify the effect of *Nkcc1* knockdown, we used an increase in the threshold of the auditory brainstem response (ABR) as a functional readout of hearing loss. As expected, *Actin*-tTS::*Nkcc1* ^tetO/tetO^ mice at P35 raised without DOX had a significantly elevated mean ABR threshold (hearing loss) compared with controls (Fig. 3f). Specifically, for *Actin*-tTS::*Nkcc1* ^tetO/tetO^ mice (n = 12) raised without DOX, the sound pressure level (SPL) at 8, 12, and 24 kHz was 83.0 ± 20.0, 82.4 ± 21.8, and 91.8 ± 18.6 dB, respectively, and that for controls (n = 6) was 25.6 ± 13.9, 32.7 ± 16.7, and 46.3 ± 15.6 dB, respectively (*p* = 0.0001, 0.0003, and 0.0002, Mann-Whitney test). These results validated that tTS-mediated *Nkcc1* knockdown in transgenic mice was functional.

In summary, the combined results showed that the FAST system–based, tTS-mediated *Nkcc1* knockdown was quantitatively, qualitatively (histologically), and functionally relevant for use in hearing studies. Hereafter, to simplify discussion, we use the term WL-KD mice to refer to *Actin*-tTS::*Nkcc1* ^tetO/tetO^ mice raised their whole lives without DOX.

### Inducible and reversible *Nkcc1* knockdown in the DOX-regulated TetO system

**Figure 4:**
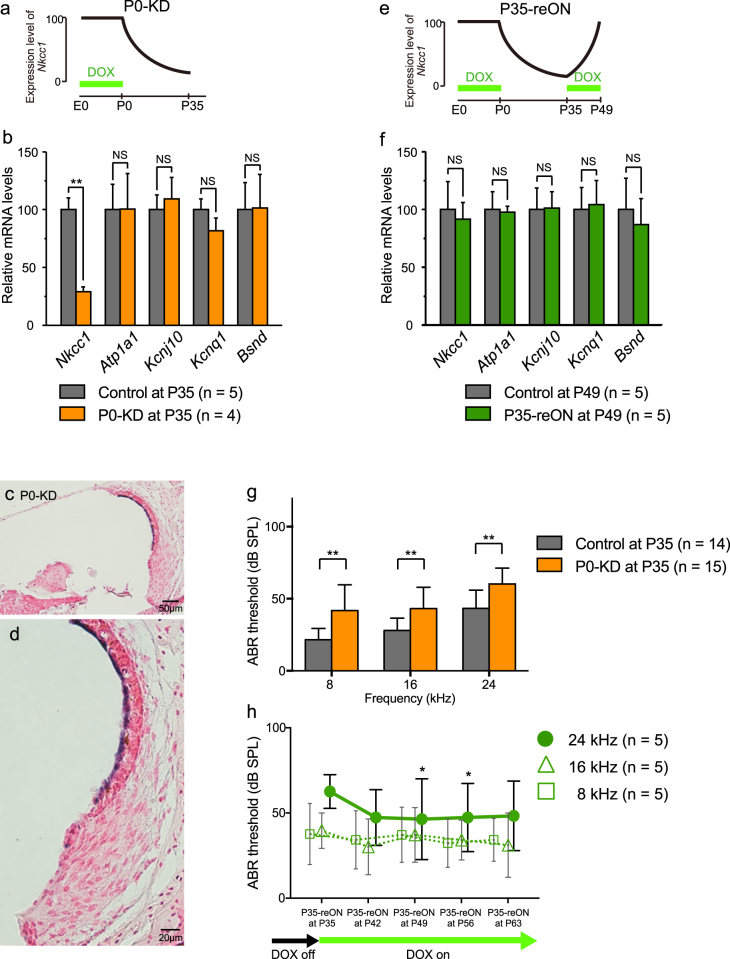
Validation of temporal control and reversibility of *Nkcc1* transcription. (**a**) The diagram shows that removal of DOX from mother’s diet suppresses *Nkcc1* expression in P0-KD progeny during lactation. (**b**) qRT-PCR analysis revealed DOX regulation of *Nkcc1* mRNA expression. At P35, cochlear *Nkcc1* transcription is decreased in P0-KD mice vs. controls in comparison with other K^+^ circulation molecules as labeled. **Significant difference at *p* < 0.01 (Mann-Whitney test). NS: no significant difference (Mann-Whitney test) in P0-KD mice at P35. (**c**) *In situ* hybridization reveals that *Nkcc1* mRNA expression was confined to the endolymphatic luminal side of the SV. (**d**) Magnified view of the image in (**c**). (**e**) The diagram reveals that, at 2 weeks post-administration of DOX in feed, *Nkcc1* mRNA expression recovered fully and specifically in P35-reON mice at P49. (f) No significant difference in the expression of *Nkcc1* in P35-reON and controls or vs. other K^+^ circulation molecules. NS: not significant (Mann-Whitney test) in P0-KD mice at P35. (**g**,**h**) Measure of ABR thresholds as readouts of hearing function. (**g**) *Nkcc1* knockdown resulted in cochlear dysfunction in P0-KD mice with significant hearing loss (higher ABR thresholds) at three different frequencies, although it was milder than that observed in WL-KD mice. **Significant difference at *p* < 0.01 (Mann-Whitney test). (**h**) ABR thresholds at 24 kHz significantly decreased for P35-reON mice, which indicates hearing recovery at high frequency (coincident with the recovery of *Nkcc1* transcription shown in panel f). *Significant difference at *p* < 0.05 (Dunn’s multiple comparison test).

One of the technical advantages of tTS-mediated gene knockdown is the temporal control of gene function via the absence or presence of DOX. To validate the versatility of *Nkcc1* knockdown *in vivo*, we examined the outcomes of postnatal knockdown. We fed pregnant mice DOX chow and then switched them to normal chow at the time of delivery to initiate *Nkcc1* knockdown in the pups from postnatal day zero (P0-KD) through adulthood, as DOX would not be available in the mothers’ milk, and the pups were maintained on normal chow after weaning at P21 (Fig. 4a). At P35, adult P0-KD mice did not show any gross abnormality in body weight or posture. The average body weight of controls (n = 5) and P0-KD mice (n = 4) was 12.6 ± 2.2 g and 12.6 ± 2.0 g, respectively (*p* = 0.7302, Mann-Whitney test). However, the level of *Nkcc1* mRNA in the P35 cochlea of P0-KD mice was significantly decreased to 30.1% of that in controls (*p* = 0.0079, Mann-Whitney test) (Fig. 4b). As with the WL-KD mice, the mRNA levels of other K^+^ circulation molecules in P0-KD mice did not decrease significantly (Fig. 4b; compare with Fig. 3a), which confirmed that *Nkcc1* knockdown was specific.

We analyzed the localization of *Nkcc1* expression in the cochlea by *in situ* hybridization. *Nkcc1* knockdown induced a uniform reduction in the *Nkcc1* signal throughout the cochlea (Fig. 4c,d). At ≥P35, *Nkcc1* mRNA in P0-KD mice was undetectable in the SLig, the Lim, and the SGN compared with moderate levels in the controls (compare with Fig. 3b,c). In the SV, *Nkcc1* mRNA level was high in the controls and still detectable in the P0-KD mice. The *Nkcc1* mRNA labeling was confined to the endolymphatic luminal side of the SV, indicating a lesser presence around the nuclei of marginal cells, which are lined up at the luminal side.

The striking feature of this gene knockdown system is that the knockdown is reversible by renewed DOX administration (Figs 2, 4e). To validate reversible *Nkcc1* knockdown, we gave DOX-containing chow to adult P0-KD mice at P35 that had been fed DOX-free chow for 5 weeks from P0, the delivery date. We named these mice P35-reON and examined *Nkcc1* expression in their cochlea. We isolated total RNA at P49 from P35-reON mice and controls (n = 5 for both groups) and found that the *Nkcc1* mRNA level returned to a level comparable to that of controls, which confirmed a full recovery of *Nkcc1* expression in these mice after 2 weeks (Fig. 4f). The levels of the other K^+^ circulation molecules in these mice did not change significantly.

### Reversibility of hearing loss in the DOX-regulated tTS-tetO system

To determine whether the observed postnatal *Nkcc1* knockdown affected cochlear function, we monitored ABR thresholds. P0-KD mice at P35 had a significantly elevated mean threshold, but the associated hearing loss was less severe than in WL-KD mice (Fig. 4g). Specifically, in adult P0-KD mice (n = 15), SPL at 8, 12, and 24 kHz was 41.8 ± 17.9, 43.2 ± 14.8, and 60.2 ± 11.0 dB, respectively, compared with 21.5 ± 7.8, 28.0 ± 8.6, and 43.3 ± 12.6 dB for adult controls (n = 14; *p* = 0.0006, 0.0008, and 0.0015, respectively, Mann-Whitney test). These results demonstrated that the DOX-regulated knockdown system induced target gene knockdown and a detectable functional change of the cochlea although the extent of *Nkcc1* knockdown was less than that observed in WL-KD.

Using DOX-inducible gene manipulation, we tested whether the hearing loss caused by postnatal *Nkcc1* knockdown was reversible. ABR thresholds of mice at P35 measured 62.5 ± 10.2 dB SPL at 24 kHz. At 2 and 3 weeks after renewal of DOX administration, the mean thresholds significantly decreased (hearing improved) to 46.0 ± 24.1 and 48.0 ± 20.7 dB SPL in P35-reON mice at P49 and P56, respectively (*p* = 0.0204 at P49 and P56, Dunn’s multiple comparisons test; Fig. 4h). However, ABR thresholds at other frequencies did not change significantly. These results indicated that the reactivation of *Nkcc1* expression partially rescued the hearing loss.

### Histopathological evidence of irreversible and reversible hearing loss upon *Nkcc1* knockdown

**Figure 5:**
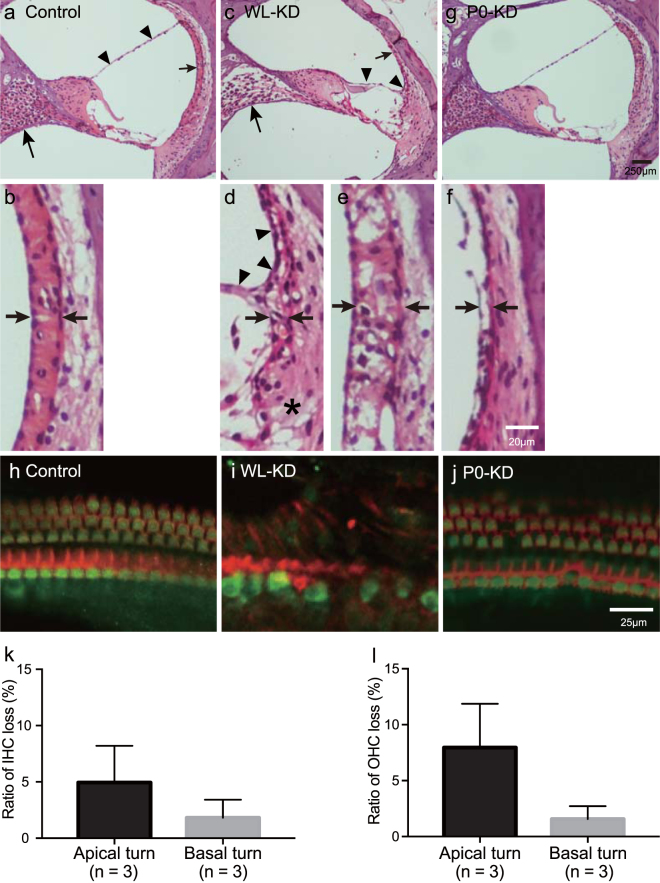
Changes in cochlear structure induced by DOX regulation of *Nkcc1* expression. (**a**–**g**) Cochlear sections from mice at P35 observed with hematoxylin and eosin staining. (**a**,**b**) Cochlear structure in a control mouse. (**a**) Low-magnification view of cochlea from controls. Arrowheads: Reissner’s membrane; smaller arrow: SV region; larger arrow: SGNs. (**b**) Higher magnification view of cochlea of control. Arrows: point to the normal width and ordered structure in the control SV. (**c**) In one-third of WL-KD mice, Reissner’s membrane (arrowheads) adhered to the SV (smaller arrow), the endolymphatic space was collapsed, and SGNs (larger arrow) were sparse. (**d**–**f**) Higher magnification views of the variable morphology of SVs of WL-KD mice. The changes in the LW were not same among all mice. (**d**) In one-third of WL-KD mice, the SV (arrows) and the type II fibrocyte region (*) were atrophic and the endolymphatic space was collapsed. (**e**) In other mice, the acellular space was wider. Arrows point to the increased width and vacuolar-filled structure in the SV. (**f**) Atrophy of the SV was conspicuous even though the endolymphatic space was not collapsed. Arrows point to the empty narrowed structure in the SV. (**g**) In P0-KD adult mice, the cochlear structure did not differ from that of adult controls. (**h**–**j**) Surface preparations of cochleae from adult mice at P35. Phalloidin staining of actin (red) and Myo7a staining (green) were used to outline HCs. (**h**) Control mice had three regular rows of outer HCs and one row of inner HCs. (**i**) WL-KD mice showed degeneration of the organ of Corti and loss of HCs; remaining HCs were disorganized. (**j**) P0-KD mice showed only mild HC loss consistent with their partial hearing recovery. (**k**,**l**) IHC and OHC loss was considerably less in the basal turn than the apical turn in P0-KD mice.

tTS-mediated *Nkcc1* knockdown yielded irreversible and reversible hearing loss in WL-KD and P0-KD mice, respectively, as measured by changes in ABR thresholds. We next investigated the histopathological factors that correlated with irreversible hearing loss. Compared with controls (Fig. 5a,b), one-third of WL-KD mice at P35 (4/12) showed collapse of the endolymphatic space (Fig. 5c), and the SV was highly degenerated (Fig. 5c–f). Even in WL-KD mice without collapse of the endolymphatic space (8/12), vacuolization or atrophy of the SV was conspicuous (Fig. 5e,f). In surface preparations, compared with controls (Fig. 5h) all WL-KD cochleae (randomly chosen; n = 5) showed massive HC loss (Fig. 5i). Generally, SGNs degenerate and are lost as a consequence of the HC loss^30^. In WL-KD mice at P35, the SGN density (n = 7; 3.5 ± 1 SGN/1,000 μm^2^) was significantly lower than that of controls (n = 5; 4.7 ± 0.9 SGN/1,000 μm^2^) (*p* = 0.0089, Mann-Whitney test; Supplementary Fig. S2). These histological findings were consistent with profound and irreversible hearing loss.

In contrast, in cross sections and surface preparations of cochleae from P0-KD mice at P35 (Fig. 5g,j), the size of the endolymphatic space, the SV, and the SLig appeared normal. The loss of HC was mild (3/3). Counting the number of HCs in surface preparations revealed that the HC loss tended to be milder in the basal turn, which mediates high-frequency hearing (n = 3; inner HC loss and outer HC loss = 1.8 ± 1.6% and 1.6 ± 1.2%, respectively), than the apical turn, which mediates low-frequency hearing (n = 3; inner HC loss and outer HC loss = 5.0 ± 3.3% and 8.0 ± 3.9%, respectively) (Fig. 5k,i), although this difference did not reach statistical significance (*p* = 0.2000 and 0.1000 for inner HC loss and outer HC loss, respectively, Mann-Whitney test). At P35, the SGN densities did not differ significantly between P0-KD (n = 5; 4.7 ± 0.6 SGNs/1,000 μm^2^) and controls (n = 6; 4.3 ± 0.5 SGNs/1,000 μm^2^) (*p* = 0.2468, Mann-Whitney test). These relatively preserved structures, especially at the basal turn, are consistent with the partial recovery of hearing loss at high frequency that we detected in P0-KD mice and correlated with DOX-induced reactivation of *Nkcc1* transcription.

## Discussion

We found that *Nkcc1* transcription was strongly inactivated in the entire body and throughout the lifetime of WL-KD mice; therefore, the degree and the nature of the loss-of-function phenotypes induced by manipulation of the tTS-tetO system by DOX were similar to those found in null mutant knockout mice (*Nkcc1* ^−/−^ mice)^18–20^. In addition, specific anatomical and functional phenotypes were shared with those observed in knockout mutants, namely, collapse of endolymphatic space, degeneration of the SV, and profound hearing loss. In these assays, the results for WL-KD mice were similar to those for conventional *Nkcc1* ^−/−^ null mutant mice.

However, the penetrance of the anatomical phenotypes was not as high in the WL-KD mice as in the null mutants. For example, in WL-KD mice, collapse of the endolymphatic space was observed in only one-third of the mice. In three past reports on *Nkcc1* ^−/−^ mice^18–20^, all described the endolymphatic collapse without rupture of the Reissner’s membrane but did not specify the penetrance of the phenotype. Flagella *et al*.^20^ alone reported occasional rupture of the Reissner’s membrane, which was attributable to excessive tension as it was pulled inwardly against the LW owing to collapse of the endolymphatic space, in addition to the collapse without membrane rupture. Two of the three publications described atrophic thinning of the SV^18,19^, and the other reported the widened intercellular spaces between the marginal cell layer and deeper layers of the SV^20^, all of which may be a consequence of loss of the high osmolality/volume–sensitive molecule, NKCC1^21^. We observed all of these changes in the WL-KD mice. The incomplete suppression of *Nkcc1* transcription in the WL-KD mice likely caused the phenotypic variation. The variability of morphology that we observed in these mice may be a phenomenon similar to that typically encountered in clinical medicine, e.g., patients with hearing loss from the same genetic abnormality do not necessarily have the same hearing levels.

P0-KD mice at P35 showed mild but significant hearing loss at all frequencies with little HC loss. Generally, HC loss causes irreversible hearing loss, but the high-frequency hearing loss was improved after the rescue of *Nkcc1* mRNA expression. Thus, the model strongly suggests that reduced *Nkcc1* mRNA level caused reversible hearing loss. Indeed, *Nkcc1* ^+/−^ mice (*Nkcc1* mRNA level, 50%) exhibited mild hearing loss without detectable associated morphological or functional abnormalities in HCs^16^.

Notably, hearing loss at 24 kHz recovered significantly after reactivation of *Nkcc1* in P35-reON mice (Fig. 4h). The highly positive EP in mammals serves to increase the sensitivity of hearing at high frequency by augmenting the electrical gradient as the driving force for K^+^ influx during transduction^15^. NKCC1 in the SV contributes to maintenance of the high EP^15^ and thus provides a plausible explanation for the hearing recovery at the high frequency; i.e., reactivation of *Nkcc1* restored the high EP potential, which then led to partial recovery of high-frequency hearing in the P35-reON mice. To restore cochlear homeostasis, this hypothesis requires that the cochlear cells retain their integrity; indeed, in support of the hypothesis, P0-KD mice at P35, especially at the basal turn, had a well-preserved cochlear structure with little HC loss.

In contrast to the restoration of hearing level at high frequency, hearing at lower frequencies did not recover. Low-frequency dominant SNHL is often seen in routine clinical practice. For example, the human genetic diseases of autosomal dominant nonsyndromic deafness1 (DFNA1), DFNA11, DFNA6/14/38, and DFNA44 exhibit low-frequency dominant SNHL^31,32^. In DFNA1, 11, 6/14/38, and 44, the responsible genes are mechanochemical or intracellular Ca^2+^ modulation proteins, namely DIAPH1, MYO7A, WFS1, and CCDC50 (Online Mendelian Inheritance in Man #124900, #601317, #600965, # 60745 and Gene ID: 1729, 4647, 7466, 152137, accessed April 21, 2017)^33^. Even though the implicated genes and functions are known, the mechanism of low-frequency dominant pathophysiology is not clear. Our results suggest that the reduction in functional NKCC1 is associated with variable hearing loss at high-frequency and irreversible low-frequency dominant SNHL. As mechanical and electrophysiological properties differ between the apical and basal turns of the cochlea^31,34^, interactions within localized areas of the cochlea between partially suppressed NKCC1 and molecules with different regional properties may be involved in the frequency-specific nature of the reversible hearing loss. For example, the difference in area of the LW between the apical and the basal turn might be a component of the mechanism for protecting HCs at the basal turn under NKCC1 suppression. The LW area of the basal turn, which mediates high-frequency hearing, is substantially wider than that of the apical turn, which mediates low-frequency hearing (Supplementary Fig. S3). This might give the basal turn more redundancy with regard to K^+^ transport capacity compared with the apical turn, and this also might result in less HC loss in the basal turn compared with the apical turn under NKCC1 suppression (Fig. 5k,l).

Considering that NKCC1 suppression can cause both reversible and irreversible hearing loss, it is notable that loop diuretics, which are widely used for attenuating extracellular fluid volume, can cause cochlear dysfunction owing to the fact that loop diuretics inhibit NKCC1^18,35^. Actually, loop diuretics can cause either temporary (or in some cases permanent) hearing loss in patients^36,37^. Therefore, each patient treated with loop diuretics should be carefully monitored for symptoms like tinnitus and hearing loss that may suggest cochlear dysfunction.

The results demonstrate that Actin-tTS::*Nkcc1* ^tetO/tetO^ mice experience inducible and reversible suppression of *Nkcc1*. However, the following technical considerations should be taken into account for further experiments. Concerning the kinetics of suppression, the DOX on-to-off silencing switch is not rapid. Once DOX chow had been replaced with normal chow, it took time (hours to days) for DOX to be excreted from the body and to reach the negligible DOX concentration that allows tTS-tetO binding^38^. Moreover, with the binding of tTS to tetO, suppression of *Nkcc1* transcription was initiated but existing protein remained. Thus, the time course of tTS-mediated knockdown depends on the half-life of the targeted protein. For example, Richardson-Jones *et al*.^39^ reported that silencing of Htr1a expression in adult Actin-tTS::*Htr1a* ^tetO/tetO^ mice started within a few days of discontinuing DOX, and the protein level became <10% of that at P28 at 4 weeks post-DOX discontinuation, which calculates to the protein half-life of ~8 days.

In the case of P0-KD mice at P35, 30% of *Nkcc1* mRNA remained, a value that was not related to the extent of the protein half-life. Less efficient transcriptional silencing of P0-KD mice may be caused by lower tTS-tetO binding efficiency. We do not understand why tTS-mediated silencing is more efficient during early embryonic periods than postnatal periods; however, such a difference is consistent with a previous report in which *Htr1b* was targeted (Actin-tTS::*Htr1b* ^tetO/tetO^ mice)^40^.

In summary, we established inducible and reversible knockdown of *Nkcc1* in postnatal mice very simply by feeding DOX-free or DOX-containing chow. Structural, electrophysiological, and molecular scrutiny of reversible hearing loss in the *Actin*-tTS::*Nkcc1* ^tetO/tetO^ mice will provide new information on the mechanisms of variable and frequency-specific SNHL.

## Methods

### General information

The experimental protocol was approved by the Animal Research Committees of Keio University School of Medicine and Niigata University School of Medicine. The experiments were carried out under the supervision of the Committees and in accordance with the Guidelines for Animal Experiments of the two universities and the Japanese Animal Protection and Management Law.

### Mouse generation and genotyping

We constructed the *Nkcc1* targeting vector by linking the following elements in tandem: the 10-kb 5′-homology arm, 3.5-kb NeoSTOPtetO cassette^28^, 1.7-kb 3′-homology arm, and the diphtheria toxin A subunit gene (Supplementary Fig. S1). The NeoSTOPtetO cassette comprised the 1.7-kb PGK-Neo, 1.3-kb STOP sequence, and 0.5-kb tetO site. The targeting vector was designed to insert the Neo-STOP-tetO cassette just upstream of the *Nkcc1* translation initiation site. We used 129 SvEv–derived embryonic cells for homologous recombination. From 80 G418-resistant clones, we obtained 3 recombinant clones. Germline-transmitted offspring were established as *Nkcc1* STOP-tetO knock-in mice (Supplementary Fig. S1). *Nkcc1* STOP-tetO mice were crossed with ROSA-Flpe mice to remove FRT-flanked Neo-STOP sequences (Supplementary Fig. S1) and establish the *Nkcc1* ^tetO^ line.

The following sets of primers were used for genotyping. The ttsP1 (5′-TTGATCACCAAGGTGCAGAG-3′) and ttsP2 (5′-CAGGGCTCTTCTCCCTTCTC-3′) were used for the *Actin*-tTS allele and yielded a band of ~400 bp. The primers Nkcc-136U (5′-TGTAGTGGCGCTGTGACTCTTTCT-3′) and Nkcc142L (5′-TCCCAGCCGTAGTAGCATCCTCTT-3′) yielded a band of ~300 bp from the wild-type allele and one of ~900 bp from the *Nkcc1* tetO knock-in allele.

### Genotypes of control and established experimental mouse lines and conditions of DOX administration

Controls: *Nkcc1* ^tetO/tetO^ littermates of *Actin*-tTS::*Nkcc1* ^tetO/tetO^ mice (from *Actin*-tTS hemizygous parents).

WL-KD mice: *Actin*-tTS::*Nkcc1* ^tetO/tetO^ mice raised for their entire lives without DOX. For this condition, pregnant and lactating *Actin*-tTS::*Nkcc1* ^tetO/tetO^ mice were fed normal chow without DOX to suppress lifetime *Nkcc1* transcription in the progeny starting from E0 to P35. There was no DOX in the systems of pregnant mice for potential transfer to embryos from stages E0–P0, in the mothers’ milk, which was the sole food source for pups from stages P0 to P20, nor was there any DOX in the chow after weaning at P21.

P0-KD mice: *Actin*-tTS::*Nkcc1* ^tetO/tetO^ mice in which *Nkcc1* knockdown was initiated on P0. Pregnant mice that had been fed DOX chow were switched to normal chow at the time of delivery. Thus, DOX was not available to the newborns in the mothers’ milk, and these mice were maintained on DOX-free chow after weaning at P21.

P35-reON mice: At P35, adult P0-KD mice were switched to DOX-containing chow after having been reared without DOX for 5 weeks. Specifically, from P0 to P20, pups received DOX-free milk from mothers; from P21-P35, mice were fed DOX-free chow; from P35 and later, mice were fed DOX-containing chow.

Definition of stages: E0 up to P0: embryo, newborn, and pup, defined by each mother’s first day of pregnancy to date of delivery; P0 to P20: newborns and pups fed with mother’s milk; P21 and up: adult mice fed with chow ± DOX.

### qRT-PCR

In WL-KD and P0-KD mice, cochlear total RNA was collected at P35. In P35-reON mice, cochlear total RNA was collected at P49 at 2 weeks after DOX re-administration. RNA was extracted using NucleoSpin RNA XS (Macherey-Nagel). cDNA was synthesized using M-MLV Reverse Transcriptase (Promega) and oligo-(dT) primers. qRT-PCR was performed on a LightCycler Nano (Roche) using FastStart essential DNA Probes Master kit and Universal ProbeLibrary Set Human (Roche). Sequences for primers were designed at the Universal ProbeLibrary Assay Design Center of Roche (https://qpcr.probefinder.com/organism.jsp) and were as follows. *Nkcc1* forward (f): 5′-tgcgagaaggtgcacaatac-3′, *Nkcc*1 reverse (r): 5′-tgtttggcttcatacgacca-3′; *Atp1a1* f: 5′-cccttaggatgtatcccctca-3′, *Atp1a1* r: 5′-caaagatgagaagggagtaggg-3′; *Kcnj10* f: 5′-tgtagaactggggttgactcg-3′, *Kcnj10* r: 5′-aatccaggatgggtgcag-3′; *Kcnq1* f: 5′-aggggagacactgctgacc-3′, *Kcnq1* r: 5′-agtactgcatgcgcctgat-3′; *Bsnd* f: 5′-catggtgatcgggggtgt-3′, *Bsnd* r: 5′-gagtctgctgggacaaag-3′; *Gapdh* f: 5′-agcttgtcatcaacgggaag-3′, *Gapdh r*: 5′-tttgatgttagtggggtctcg-3′. Expression was normalized to that of *Gapdh* and quantified based on the 2^−ΔΔCT^ calculation. The data are displayed as 2^−ΔΔCT^ and 2^−ΔΔCT^ ± SEM for each group and each gene. Statistically significant differences were evaluated between KD mice and control mice using the unpaired Student’s *t* test.

### *In situ* hybridization

Mice were deeply anesthetized with ketamine/xylazine and intracardially perfused with 0.1 M sodium phosphate buffer (PB, pH 7.4) containing 4% (w/v) paraformaldehyde. Fixed crania containing the temporal bones were collected, and bone decalcification was accomplished by immersion in 0.5 M EDTA (pH 7.5) for 7 days. Bone containing cochlea was embedded in OCT compound, frozen, cut at 10 µm thickness, mounted on a glass slide, and subjected to *in situ* hybridization^41^. Briefly, cryosections were treated with proteinase K (40 g/mL). Sections were washed, acetylated, and incubated with digoxigenin-labeled mouse *Nkcc1* cRNA probe. The *Nkcc1* cRNA probe covers nt 3899–6451, (NM_009194; 6520 bp; coding region is 130–3750 bp). Sections were washed in buffers with serial differences in stringency and incubated with an alkaline phosphatase–conjugated antibody against digoxigenin (1:5,000; Roche). The cRNA probes were visualized with freshly prepared colorimetric substrate (NBT/BCIP; Roche), and nuclear fast red (Vector Labs) was used for counterstaining.

### Auditory brainstem response recording

ABR thresholds to tone burst stimulus at 8, 16, and 24 kHz were measured^42^. Briefly, mice were anesthetized by intraperitoneal injection of ketamine and xylazine for recording. ABR waveforms were recorded for 12.8 ms at a sampling rate of 40,000 Hz using 50–5,000 Hz bandpass filter settings. Waveforms from 1,024 stimuli at a frequency of 9 Hz were averaged. The thresholds of ABR were determined using a 5-dB SPL minimum step size down from a maximum amplitude until distinct ABR wave patterns were no longer recognized. In WL-KD mice and P0-KD mice, thresholds were measured at P35. In P35-reON mice, thresholds were measured every week after P35 for 4 weeks. Numbers of analyzed mice are shown in the Results.

### Morphological analysis with hematoxylin and eosin staining

After cardiac 4% paraformaldehyde/PB perfusion as described in *in situ* hybridization, the inner ears were harvested in 0.1 M PB saline and immersed in 4% paraformaldehyde/PB. Fixed inner ears were collected and decalcified in 0.5 M EDTA for 7 days. Paraffin sections of 5 μm thickness were made in the horizontal plane parallel to the modiolus. For morphological analysis, they were stained with hematoxylin and eosin. For the analysis, more than three mice were randomly chosen in each study group.

SGN density was assessed in the paraffin sections. Images of upper- and lower-middle turns in each section were taken to include the entire Rosenthal’s canal. SGN density in Rosenthal’s canal was measured using NIS-Elements (Nikon).

### Surface preparations of HCs in the organ of Corti

Surface preparations were made at P35^42^. For immunolabeling, preparations were incubated overnight at 4 °C with anti-myosin VIIa (1:500; Proteus Biosciences) followed by rhodamine-phalloidin (1:100; ThermoFisher) and Alexa488-conjugated anti-rabbit secondary antibody (1:100; ThermoFisher) for 60 min at room temperature. Actin was labeled by rhodamine-phalloidin. Myosin VIIa is a specific marker for HCs in the inner ear^43^. HCs were visualized by laser-scanning confocal microscopy (Zeiss LSM 710.). HC loss in the apical turn and basal turn was counted manually. Two or more microscopic fields at magnification 20x for each turn and each mouse were used for counting.

### Statistical analysis

All data are presented as the mean ± standard deviation except for the qRT-PCR data, which are mean ± standard error. Statistical tests are indicated in the Results. A *p* value of <0.05 was considered significant. All statistical analyses were carried out using Prism (GraphPad Software, Inc.).

### Data availability

The datasets generated and/or analyzed during the current study are available from the corresponding authors.

## Electronic supplementary material

Supplementary information
