# Supplementary material for: Time-controllable Nkcc1 knockdown replicates reversible hearing loss in postnatal mice
Source: Sci Rep. 2017 Oct 19;7:13605. doi: 10.1038/s41598-017-13997-7 (PMC5648887; doi:10.1038/s41598-017-13997-7)
Supplement: Supplementary file 1 — Supplementary information [file 41598_2017_13997_MOESM1_ESM.pdf]

Supplementary information

Time-controllable *Nkcc1* knockdown replicates reversible hearing loss in postnatal mice

Takahisa Watabe[1], Ming Xu[2], Miho Watanabe[3], Junichi Nabekura[4], Taiga Higuchi[5], Karin Hori[5], Mitsuo P. Sato[5], Fumiaki Nin[5], Hiroshi Hibino[5,6], Kaoru Ogawa[1], Masatsugu Masuda[1,7]*, Kenji F. Tanaka[2]*

***Corresponding authors:**

Masatsugu Masuda

Tel: +81-422-42-5968.

Email: masoeur13@mac.com

Kenji F. Tanaka

Tel: +81-3-5363-3934.

Email: kftanaka@keio.jp

Supplementary Figure S1

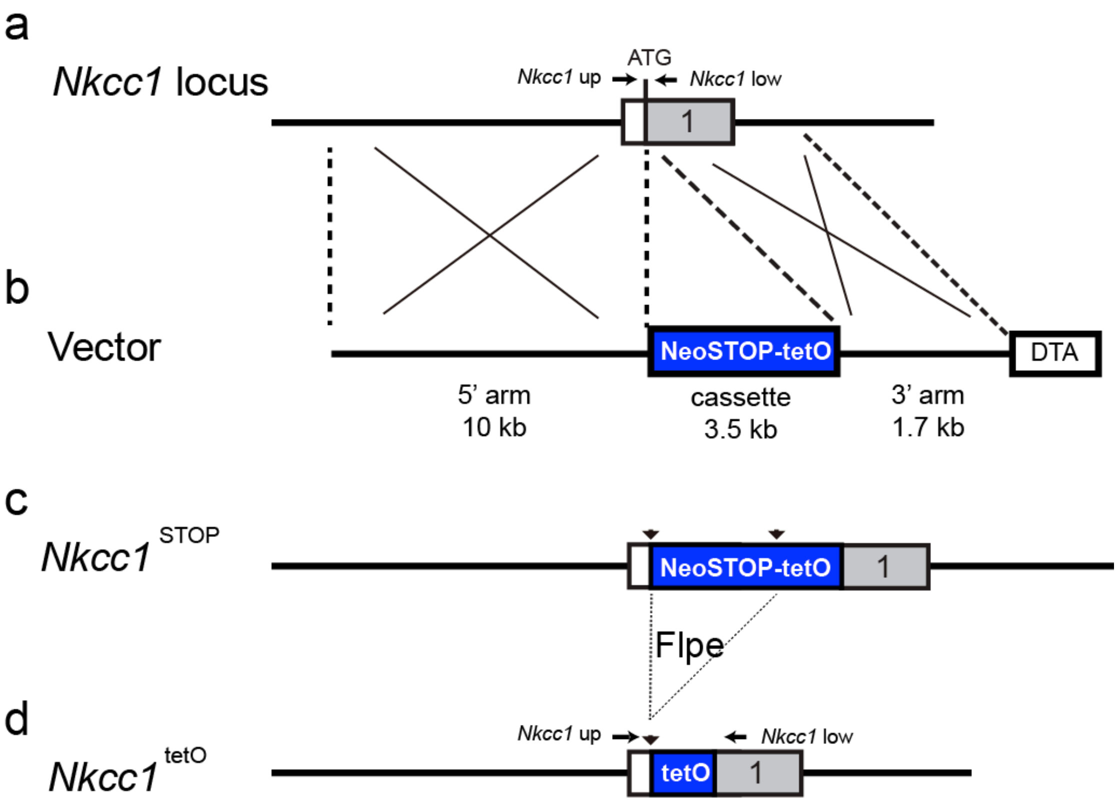

Supplementary Fig. S1. Diagram depicting the strategy for inserting the Neo-STOP-tetO cassette into the *Nkcc1* locus. (a, b) Relationship between the *Nkcc1* locus and targeting vector. (a) Starting *Nkcc1* locus. ATG indicates the translation initiation site of *Nkcc1* in exon 1. White box: 5′-untranslated region; gray box: coding region. Arrows show sites for genotyping primers (*Nkcc1* up and low). (b) *Nkcc1*^{tetO} targeting vector that carries the Neo-STOP-tetO cassette knock-in allele. Lengths of different elements are shown below the corresponding regions. (c)

Nkcc1^{STOP}:Neo-STOP-tetO cassette knock-in allele that carries the FRT-flanked Neo-STOP target of flippase, Flpe. This insert was excised between the two arrowheads by crossing with Flippase mice (Flpe). Arrowheads: FRT sites. (d) Final *Nkcc1*-tetO construct. Arrowhead: Remnant of FRT sites.

Supplementary Figure S2

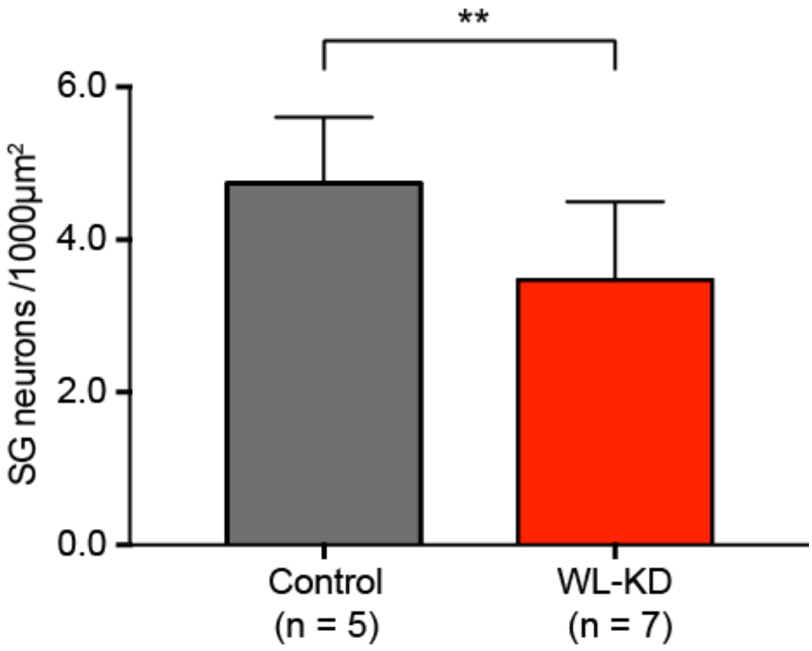

Supplementary Fig. S2. Statistical analysis of SGN density in WL-KD mice. WL-KD mice had a significantly lower density of SGNs than controls. **Significant difference at $p < 0.01$ with the Mann-Whitney test.

Supplementary Figure S3

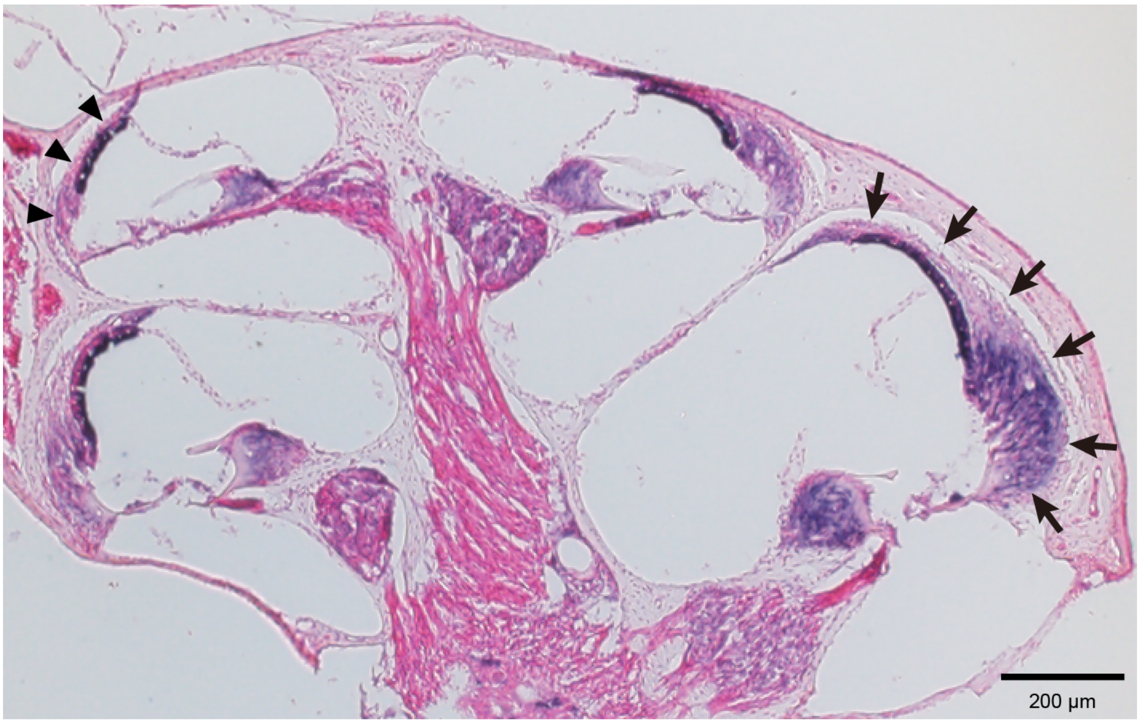

Supplementary Fig. S3. Difference in the area of the LW between the apical and basal turn. Shown is a cross-section of a whole cochlea of a control mouse subjected to *in situ* hybridization specific for *Nkcc1* mRNA. The LW is substantially wider in the basal turn (arrows), which mediates high-frequency hearing, than in the apical turn (arrowheads), which mediates low-frequency hearing.
